# Developing a Computerized Adaptive Test to Assess Stress in Chinese College Students

**DOI:** 10.3389/fpsyg.2020.00007

**Published:** 2020-02-07

**Authors:** Xueyin Tian, Buyun Dai

**Affiliations:** ^1^School of Psychology, Jiangxi Normal University, Nanchang, China; ^2^Department of Psychology, School of Social Development and Public Policy, Fudan University, Shanghai, China

**Keywords:** stress, computerized adaptive test, item response theory, item bank, measurement precision

## Abstract

Stress is among the most prevalent problems in life; thus, measurement of stress is of great importance for disease prevention and evaluation. This work aims to develop a computerized adaptive test (CAT) application to measure stress (CAT-S) based on item response theory (IRT). Two types of analyses were performed. The first analysis was to meet the psychometric requirements of the CAT-S. A Paper and Pencil (P&P) test involving 226 items was developed based on eight stress-related scales, and 972 Chinese college students completed the test. The first seven scales were used to build the item bank, and the last scale (i.e., the Perceived Stress Scale, PSS) was used to determine the convergent validity of the CAT-S. With some statistical considerations, such as item fit, discrimination, differential item functioning (DIF), and the assumption of unidimensionality, the final item bank comprised 93 items. The second analysis was to simulate the CAT adaptively using the existing item response. A Bayesian method called Expected a Posterior method (EAP) was used to estimate θ. For the item selection strategy, the greatest item information was considered at each step. The stopping rule was determined by the fixed length (10, 11, 12, …, 20, and 93) or the prespecified level of measurement precision (standard errors of 0.3, 0.4, 0.5, 0.6, 0.7, and 0.8). Finally, the criterion validity was tested by using PSS as a criterion and analyzing the effect of CAT-S diagnosis with a receiver operating curve (ROC). The results showed that (1) the final stress item bank had good quality based on the psychometric evaluation, (2) the CAT-scores were highly correlated with the scores of the final item bank, (3) the scores of the P&P form of PSS were correlated with those of the CAT-S (*r* > 0.5), (4) the value of the area under the ROC curve (AUC) was greater than 0.7 under each stopping rule, and (5) the CAT-S needed only a small number of items to obtain a highly precise measure of stress. Therefore, the CAT-S presented the theoretically expected advantages, which enabled a rapid, accurate, and efficient dynamic and intelligent measurement of stress.

## Introduction

Lazarus and Folkman (1984) proposed that stress was considered as the interaction between humans and the environment and used “person–environment fit” to explain it. When people face a potentially stressor, the intensity of the stress they experience depends on the evaluation of the event (appraisal) and their personal resources (coping). Good human adaptation to the environment leads to lower stress, and poor adaptation to the environment leads to higher stress. An increasing body of evidence has proven that high stress can lead to a variety of diseases, including physical and mental diseases, such as coronary heart disease, mental illness, or other occupational diseases (Motowidlo et al., 1986). Stress is a causal factor for poor health when adaptational demands exceed the body’s ability to resist stress (Amirkhan, 2012). A moderate rather than excessive amount of stress can actually be beneficial (Yerkes and Dodson, 1908). Therefore, it is essential to assess the severity of stress for disease prevention and evaluation.

To date, stress has been evaluated primarily with self-report scales, most of which were developed with classical test theory (CTT). Many well-known stress scales have been established, such as the Perceived Stress Scale (PSS) (Cohen et al., 1983), the Stress Overload Scale (SOS; Amirkhan, 2012), and the Depression Anxiety Stress scale (DASS; Chan et al., 2012). However, these scales have some drawbacks. First, these scales only model the total score; hence, they cannot quantify the quality of items. Second, these scales cannot be used for comparison among different test score systems. Third, the item difficulty to endorse and the participants’ stress level are not on the same scale. Finally, a large number of items are needed to meet the high measurement precision of the construct based on CTT.

To address the problems mentioned above, researchers have applied computerized adaptive testing (CAT) based on item response theory (IRT) to improve CTT, which can improve efficiency, balance measurement precision, and reduce respondent burden. There are some advantages with IRT, which provides a more comprehensive measurement framework for testing than does CTT (Edelen and Reeve, 2007). Furthermore, IRT provides the most appropriate items for given participants, which is a vital component of CAT. The system selects items automatically based on what it knows from previous items.

Due to the adaptive nature of the test, the participants receive different item sets. Generally, IRT-based CAT algorithms consist of five components: calibrated item bank, starting level, item selection, scoring method, and stopping rule (Smits et al., 2011). Furthermore, CAT makes it possible to use fewer items to achieve higher measurement precision, which can also reduce floor and ceiling effects (Embretson and Reise, 2000).

In previous studies, CAT was initially designed for cognitive tests (Wainer, 2000). A variety of CAT procedures have been used recently to assess personality or attitude (Yang, 2017; Bagby and Widiger, 2018). In addition, CAT has attracted much more attention in the field of quality of life issues, such as depression (Loe et al., 2017) and anxiety (Gibbons et al., 2017). However, the level of stress has seldom been studied in CAT.

In recent years, Chinese students’ mental health issues have become more serious, mainly because of various types of school-related stress (Duan, 2016). Due to the need for clinical research in China, we performed this research in the Chinese cultural environment. It is true that a certain level of stress may promote progress, but excessive stress can endanger health. Considering the shortcomings of CTT and the advantages of CAT, this study aimed to provide a new stress assessment technique by using CAT with a group of college students as the measurement sample. The purpose is to assess the actual stress levels of college students accurately, to gain an in-depth understanding of the psychological stress of college students, and to help them reduce psychological crises caused by stress. With CAT, the number of items is reduced while ensuring the accuracy of the test. Reliable evaluations can be obtained with participants with only a small number of items, which saves time and resources and facilitates accuracy, efficiency, and speed (Embretson and Reise, 2000).

Through the data analysis, this study develops a CAT of stress that mainly consists of the following two parts: (1) the construction of the computerized adaptive test for stress (CAT-S) item bank and (2) a real data CAT-S simulation study. We select a certain number of stress-related scales to form an initial item bank, analyze the psychometric characteristics of the item bank, obtain an adaptive complete item bank, and simulate the CAT with real data. Finally, we compare the application conditions under different degrees of measurement precision and explore the effectiveness of the CAT-S, such as its reliability and validity.

## Materials and Methods

### Participants

The overall sample consisted of 972 Chinese University students (38.0% males, 62.0% females) with an average age of 19.12 years (SD = 1.39, ranging from 16 to 25) from different regions (49.3% urban, 50.7% rural) and majors (35.6% arts, 58.3% science, and 6.1% other). Students volunteered to participate.

### Construction of the Item Bank for Stress

#### Measures

The Paper and Pencil (P&P) test comprises 226 items among eight Chinese version scales, including the DASS, SOS, College Student Stress Scale (CSSS; Li and Mei, 2002), Psychological Stress Feeling Scale for College Students (PSFS; Zhang et al., 2003), Psychological Stress Measurement Scale (PSMS)^1^, Psychological Stress Test (PST)^2^, Stress Scale for College Students (SSCS)^3^, and PSS. The first seven scales were used to build the initial item bank, and the final scale was used to determine the convergent validity of the CAT-S, which referred to the methods of previous scholars (Smits et al., 2011). All the scales were regarded as indicating the severity of stress in the previous month, and high mean scores reflected severe stress.

The DASS consists of 21 items with three factors, and we used only seven items related to stress. Items are scored on a Likert rating scale from 0 (did not apply to me at all) to 3 (applied to me very much). The alpha coefficient for the Chinese version of the DASS was 0.93 in a Chinese sample (Chan et al., 2012), and it is 0.81 in the current study.

The CSSS measures stress with 30 questions. We used 24 items among the personal hassle and academic hassle factors. Items are scored from 0 (no stress) to 3 (a lot of stress). CSSS featured good psychometric characteristic with a high coefficient alpha (0.91) in a Chinese sample (Li and Mei, 2002), and Cronbach’s alpha is 0.92 in the current study.

The SOS is the most recently published general stress measure, with a five-point Likert-type scale ranging from 1 (not at all) to 5 (a lot). The Chinese version of the SOS consists of 22 items, and the coefficient alpha was 0.94 in a Chinese sample (Su and Guo, 2014), which indicated good reliability. In the present study, the coefficient alpha of the SOS is 0.94.

The PSFS consists of 62 items. Participants were asked to indicate their feelings on a five-point Likert scale from 1 (never) to 5 (very strong). The PSFS possessed good reliability (alpha = 0.86) in a Chinese sample (Zhang et al., 2003), and the value is 0.97 in the present study.

This study also used three stress-associated scales from the Internet, including the PSMS (23 items), PST (50 items), and SSCS (21 items). In the current study, Cronbach’s alpha values for the three scales are 0.91, 0.95, and 0.89, respectively.

Finally, the PSS, a 14-item scale for measuring non-specific perceived stress, has become one of the most widely used psychological instruments. Items are scored from 0 (never) to 4 (very strongly agree). Individuals who obtain a total score greater than 28 are considered to have high stress. The PSS has been found to have adequate validity (Lee, 2012), and the internal consistency of this measure in the current study is acceptable (Cronbach’s alpha = 0.80). In addition, the three scales (SOS, PST, and SSCS) each contained a lie detection item, which was only used to filter participants and did not participate in the subsequent item bank construction process.

#### Unidimensionality

Above all, a one-dimensional model would be checked. Two conditions must be verified to assume unidimensionality: First, the first factor should account for at least 20% of the test variance (Reckase, 1979). Second, the ratio of the variance of the first factor to that of the second factor is higher than 4 (Reeve et al., 2007).

To confirm the unidimensionality of the selected items from seven scales, we first conducted a one-factorial exploratory factor analysis (EFA) to eliminate items with factor loadings less than 0.40 at a time (Wu et al., 2019). This approach was supported by Nunnally’s (1978) conclusions, which suggested that factor loadings smaller than 0.30 should not be taken seriously and that factor loadings smaller than 0.40 can easily be overinterpreted. We again conducted one-factorial EFA until the factor loadings for all remaining items were more than 0.4. Finally, we examined the level of interpretation of the main factor and the secondary factor on the overall test variance and calculated the ratio of these two factors. Then, we again conducted EFA with multiple factors, and the method of extracting factors was based on the rule of having a characteristic root over 1.

#### IRT Model Comparison

After the item bank meets the assumption of unidimensionality, a suitable IRT model must be selected for the parameter estimation. Some commonly used IRT models for polytomous data include the graded response model (GRM; Samejima, 1968), the generalized partial credit model (GPCM; Muraki, 1997), and the rating scale model (RSM; Andrich, 1978). To compare the accuracy of different IRT models and select the most suitable IRT model, the following three popular model fit indices were used: −2 log likelihood (−2LL), the Akaike information criterion (AIC), and the Bayesian information criterion (BIC). The three indices measured the goodness of fit of the statistical model and helped us to choose the model with the best fit. In general, smaller indices indicate better IRT model fit. The mirt R package (Chalmers, 2012) was used to carry out the model comparison and selection.

#### Local Independence

Local independence is an essential part of the IRT model, which means that item responses are not associated with each other when controlling for trait levels. To test local independence, the residual correlation was analyzed by Yen’s (1993) Q3 statistic using the mirt R package. The statistic is used to calculate the residual item scores and correlations among items under the IRT model. Cohen’s (1988) rules of thumb for correlation effect sizes suggest that Q3 values from 0.24 to 0.36 are moderate deviations and that values over 0.36 represent large deviations. Therefore, items with large deviations were eliminated from the item bank.

#### Item Discrimination

The items were chosen according to the discrimination parameters of the IRT model. The items with a discrimination ability of less than 0.7 were considered to have low quality (Fliege et al., 2005) and were removed from the item bank.

#### Item Fit

The *S*−χ^2^ statistic (Kang and Chen, 2008) was applied to test the item fit. Items with *p*-values of *S*−χ^2^ less than 0.01 were deemed to have a poor item fit (Flens et al., 2017) and were eliminated from the item bank.

#### Differential Item Functioning (DIF)

Differential Item Functioning (Embretson and Reise, 2000) analysis is used to identify systematic differences caused by group bias (the independent “group” variable was gender). In the multiscoring scale, DIF can be tested by logistic regression using the McFadden’s (1974) pseudo R^2^ method. This was identified in the lordif R package (Choi et al., 2011), and items with DIF were deleted one by one until the test had no DIF items.

### The Simulated Stress CAT (CAT-S)

To simulate the adaptive version of the stress test, a CAT program was employed in the statistical environment of R software.

### CAT-S Procedures

In the CAT procedure, the entry level was set to 0, and the Fisher information item selection method was used. The item with the greatest item information with the initial latent value was chosen as the first item. To estimate θ, a Bayesian method called Expected a Posterior method (EAP; Embretson and Reise, 2000) was used. For the item selection strategy, the greatest item information was considered at each step. The stopping rule was determined by the fixed number of items administered or the prespecified measurement accuracy. In order to reduce the burden of participants especially stressful people (Furnham, 1997), the maximum fixed number of items was set to 20 after trying a lot of item lengths. Then, some fixed-length stopping rules including 10, 11, 12, …, 20, and 93 items were applied to find a better one. To explain the rules between different precisions, the CAT was run under the required standard errors (SEs) of θ (SEs of 0.3, 0.4, 0.5, 0.6, 0.7, and 0.8). When the number of items has reached a certain amount, and even if the accuracy requirements have not been met, it is forcibly terminated. However, the specific value of “a certain number of items” should be determined based on the simulation results of the fixed-length test. Once the measurement accuracy or the maximum number of items was met, the test was terminated. In addition, an approximated value of classical reliability was provided.

#### Comparing Complete Data With the CAT Data

To prove the efficiency of the adaptive version, the CAT estimation must be similar to that of the final item bank, and the diagnosing criterion must be used to fix the item bank.

Two analyses were studied to compare the complete data and CAT data. One analysis calculated the Pearson correlations between their estimates (Cohen et al., 2009), and another method was used for criterion validity (McDonald, 1999). In this part, two criteria were set to check the results. The first criterion variable was a well-known stress scale (PSS), and concurrent validity was ensured using the Pearson correlation coefficient. The other one was a classification of high stress based on diagnosis by the PSS. The predictive utility (sensitivity and specificity) was assessed by the area under (AUC) the receiver operating curve (ROC). The “sensitivity” represents the probability that the participants are accurately diagnosed as having greater stress, while the “specificity” indicates the probability of being diagnosed as having no greater stress. In addition, the ROC curve for each stopping rule was plotted by the software SPSS 23.0. Among them, the PSS scale was used for the classification of stress, and the estimated theta in CAT-S was used as a continuous variable. AUC can evaluate the statistics for ROC curves with values ranging from 0.5 to 1. It is similar to random guessing with AUC = 0.5, and the predictive utility is perfect when AUC = 1. Besides, the predictive utility is moderate when AUC ranges from 0.7 to 0.9. The greater the AUC value, the better the diagnostic effect (Kraemer and Kupfer, 2006).

## Results

### Psychometric Evaluation of the Stress Item Bank

#### Unidimensionality

Considering the factor loadings below 0.4 in the EFA, 38 items were excluded from the initial item bank. The first factor in a principal component analysis of the polychoric correlations accounted for 30.44% of the test variance, which met the Reckase (1979) criterion of 20%. The second factor explained only 4.30% of the variance, and the ratio of variance explained by the first two factors was 7.08, which was higher than the required minimum of 4. Based on these results, the remaining item bank for stress presented a single common factor (stress).

#### IRT Model Comparison

To find a suitable IRT model to fit the dataset, three commonly used polytomous IRT models were performed in R software, including GRM (−2LL = 340315, AIC = 341973.5, BIC = 346018), GPCM (−2LL = 341498, AIC = 343156, BIC = 347201), and RSM (−2LL = 347025, AIC = 347663, BIC = 349219). Obviously, the GRM model had the best fit among these models; therefore, this model was chosen for the subsequent analysis.

#### Local Independence

Under the GRM model, local independence was tested with the Q3 statistic. Because the Q3 value was larger than 0.36, 65 items were eliminated from the item bank. The remaining item bank supported local independence.

#### Item Discrimination

Regarding item discrimination, the values of all items were over 0.8 as expected. All the items had a relatively high level of discrimination power.

#### Item Fit

The *S*−χ^2^ statistic was examined. Eleven items were excluded because the *p*-values of *S*−χ^2^ were less than 0.01. Based on these results, it was proven that all remaining items fit well with the GRM.

#### Differential Item Functioning

The “gender” variable was used to analyze DIF. Gender was divided into two levels, including males and females. According to the pseudo R^2^ method, two items had DIF for gender in the changes in R^2^ over 0.02. The remaining items showed no DIF for gender after eliminating the two items.

#### Item Parameters

The GRM parameter estimation of items is shown in Table 1. The second column of the table represents the discrimination parameters (a); among these, item 19 has the lowest value (*a* = 0.816), and item 46 had the highest value (*a* = 2.804). The average level of discrimination was 1.376, indicating that the item bank had rather higher discrimination power and a considerable level of item quality. The other columns show the estimation of the threshold parameters. Furthermore, as expected in the GRM, the value of the threshold parameters for the items was ordered from b_1_ to b_3_ (b_4_).

**TABLE 1:** Estimated GRM parameters of a 20-item example from the final stress item bank (93 items).

**Item**	**Item parameters**				
	**a**	**b_1_**	**b_2_**	**b_3_**	**b_4_**
1	0.942	–0.609	2.211	4.352	–
2	0.902	–1.761	0.812	3.387	–
3	1.120	–0.388	1.833	3.388	–
4	0.868	–0.500	2.318	4.240	–
5	0.851	–0.421	2.156	3.650	–
6	0.995	–1.154	1.604	4.030	–
7	1.196	0.226	2.584	3.986	–
8	1.016	–0.189	2.112	4.123	–
9	1.013	0.612	3.268	5.360	–
10	1.509	0.205	1.928	3.406	–
…	…	…	…	…	…
84	1.048	–0.612	1.080	3.083	4.671
85	0.899	–2.130	–0.216	1.692	3.833
86	1.186	–0.978	0.625	1.978	3.227
87	1.024	–1.086	0.600	2.045	3.271
88	1.156	–1.269	0.398	1.868	2.996
89	1.275	–0.796	0.533	1.880	3.195
90	1.513	–0.276	0.958	1.931	3.051
91	1.170	–0.445	0.884	2.177	3.257
92	1.197	0.075	1.578	2.886	4.019
93	0.904	–0.202	1.561	3.049	4.020

*Note: a is the discrimination parameter; b refers to the location parameters; the first 17 items are scored with 0, 1, 2, 3, and the last 76 items are scored with 0, 1, 2, 3, 4.*

After the previous steps, the final stress item bank comprised 93 items with acceptable quality (Table 2 presents the source of the item bank. Readers can ask the authors for the item bank).

**TABLE 2:** The source of the item bank.

**Type**	**The item length**	**The number of items and the item number (final item bank, renumbering after removing lie detection items)**
DASS	7	4 (2, 3, 5, 7)
CSSS	24	13 (1–3, 5, 6, 8, 9, 12–16, 24)
SOS	22	5 (3, 5, 11, 16, 20)
PSFS	62	33 (1, 3, 4, 6, 8, 10, 13, 15, 24, 25, 27, 31, 33–35, 38, 41, 42, 46, 48, 50–59, 62)
PSMS	23	8 (1, 4, 5, 7–9, 16, 17)
PST	50	23 (2–4, 9, 12, 13, 16, 17, 19, 23, 24, 26, 27, 30, 33, 34, 37–39, 44, 48–50)
SSCS	21	9 (4, 6–8, 10, 12, 17, 19, 21)
Total	209	93

### Stress CAT (CAT-S) Simulation

#### Characteristics of the CAT

First, under the condition of fixed number of items, when the length was set to 10 to 20 items, the average SE was between 0.245 and 0.322, and the reliability was higher than 0.890. Besides, the correlation coefficients between CAT-S and the final stress item bank were greater than 0.937. It can be seen that the precision obtained under these rules is high, the reliability is good, and it has a high correlation with the final stress item bank. In addition, the PSS has a total of 14 items, and the number of items in other scales participating in the construction of the item bank is greater than 14 questions (except for a subscale of seven questions extracted from DASS-21). Therefore, considering some factors (SE ≤ 0.3, reliability ≥ 0.9, and the length of items should not be so long, especially to take care of the emotions of high-stress subjects), 13-item was considered to be the optimal fixed length. After that, on the basis of the item length of 13, the influence of different precisions was further explored. It was found that the rule with an accuracy of 0.3 was optimal.

Table 3 shows the characteristics of the different stopping rules for the CAT-S. In addition to the defined column, the first row shows the CAT outcomes of the final stress item bank, while the first two columns show the average number of items used and the corresponding standard deviation (SD). The number of items used was clearly higher when the level of measurement precision and the SD were higher. The fourth column is the average SE of the actual θs on the basis of different levels of measurement precision. First, under all the rules of fixed length, the precision is high. Even when the item length is 10, the SE still reached 0.322. Second, when setting the length and termination precision rules at the same time, the actual SEs were expected to be smaller than those of the stopping rule. However, when the stopping rule was SE(θ) < 0.3, the average SE was slightly over 0.3. In fact, some participants may proceed through the preset maximum number of items (13) before meeting the prespecified value of SE; thus, the actual SE was higher than expected.

**TABLE 3:** Characteristics of the CAT under different stopping rules.

**Stopping rule**	**Number of items used**		**Mean SE(θ)**	**Marginal reliability**	**Correlation between CAT θ and complete test θ**
	**M**	**SD**			
None	93	0	0.156	0.974	1.000
length(item) = 10	10	0	0.322	0.890	0.937
length(item) = 11	11	0	0.309	0.898	0.940
length(item) = 12	12	0	0.300	0.905	0.947
length(item) = 13	13	0	0.290	0.911	0.951
length(item) = 14	14	0	0.281	0.916	0.953
length(item) = 15	15	0	0.273	0.921	0.958
length(item) = 16	16	0	0.267	0.924	0.960
length(item) = 17	17	0	0.260	0.928	0.962
length(item) = 18	18	0	0.255	0.931	0.964
length(item) = 19	19	0	0.249	0.934	0.966
length(item) = 20	20	0	0.245	0.937	0.969
SE(θ) < 0.3 or length(item) = 13	10.128	2.136	0.319	0.895	0.937
SE(θ) < 0.4 or length(item) = 13	6.658	2.880	0.387	0.849	0.915
SE(θ) < 0.5 or length(item) = 13	4.579	2.469	0.467	0.781	0.888
SE(θ) < 0.6 or length(item) = 13	3.520	1.714	0.528	0.719	0.865
SE(θ) < 0.7 or length(item) = 13	2.818	1.495	0.603	0.634	0.848
SE(θ) < 0.8 or length(item) = 13	2.467	0.843	0.638	0.588	0.832

*Note: None, no stopping rule (perform all items); SE, standard error; M, mean; SD, standard deviation.*

To compare the precision of CATs, marginal reliability (Green et al., 1984) was estimated under the framework of IRT. The results for marginal reliability can be found in the fifth column of Table 3, indicating a desirable level of reliability. The reliability estimates under different fixed-length rules were slightly different. When the item length was 20, the reliability was 0.937; when the item length was 10, the reliability was 0.890. However, when the fixed length was set to 13, the differences in reliability estimates were relatively large. For instance, the value was 0.895 with the SE(θ) < 0.3 level of precision and remained at 0.719 when the stopping rule was SE(θ) < 0.6. The measurement precision was influenced by the latent trait in IRT, which was not indicated by the single value for overall reliability. Instead, marginal reliability was calculated by the average reliability of all traits. The result should be a uniform distribution for test information in order to indicate an accurate estimate.

Finally, the sixth column shows the correlations between the estimated θ of the CAT and that of the final stress item bank. Clearly, all the correlations among them were relatively high. It was greater than 0.937 under all the fixed-length rule. Besides, under the condition of fixed length and termination accuracy, the degree of correlation was also acceptable. For instance, although the participants answered only 4.579 items on average when the measurement precision was set to SE(θ) < 0.5, it had a high correlation of nearly 0.888. In addition, it was obvious that the correlations were influenced by the level of measurement precision. Even with an average of 2.467 items used in the SE(θ) < 0.8 level of precision, the correlation was nearly 0.832.

#### Criterion Validity of the CAT

**FIGURE 1:**
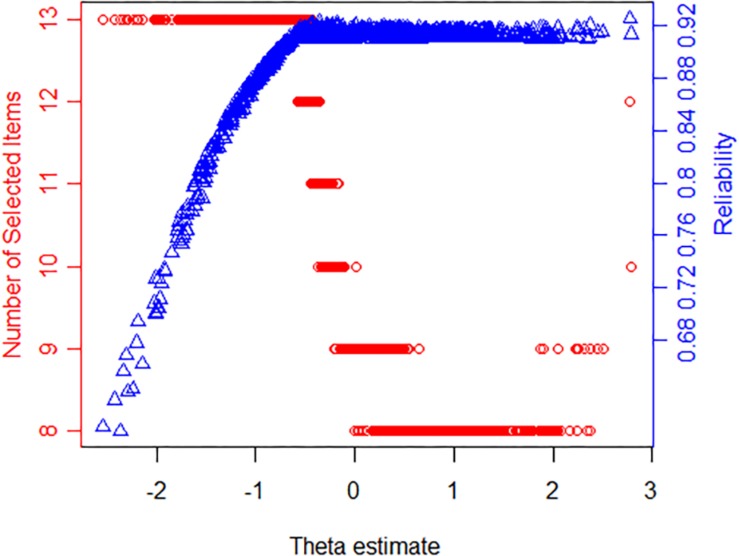
The relationship among the latent stress (θ) estimate, reliability, and the number of selected items for the stopping rule SE(θ) < 0.3 or length(item) = 13 (dots represent respondents; triangles show reliability).

The SE(θ) < 0.3 or length(item) = 13 stopping rule is considered a better termination rule. At this time, 673 participants answered no more than 13 items, accounting for 69.17% of the total number of participants. Moreover, we presented the number of items used and reliability with the estimated θ under the SE(θ) < 0.3 or length(item) = 13 stopping rule in Figure 1. As shown, the maximum number of items (13) was administered when the estimated θ was low, indicating lower reliability. The number of selected items decreased with a decline in measurement precision. Surprisingly, the reliability value was almost 0.92 under the θ estimates over about −0.5 in Figure 1. Therefore, there was a high amount of information in the θ estimate interval from −0.5 to 3, which indicated that the CAT-S was able to measure most participants except the theta estimates below −0.5.

Table 4 shows the relationship between the CAT estimates and stress-related variables. The correlation between the estimated θ in the complete data and the PSS sum score (column 1) was 0.631. Generally, this correlation decreased as the level of measurement precision or length declined. For example, when the SE(θ) < 0.5 or length(item) = 13 rule (average number of items was 4.579) was used, the correlation decreased to 0.577.

**TABLE 4:** Relationship with external criteria of the stress CAT (CAT-S) estimates under different stopping rules.

**Stopping rule**	**Perceived stress scale (*r*)**	**High stress (*AUC*)**
None: sum score	0.626	0.792
None: $\hat{\theta }$	0.631	0.793
length(item) = 10	0.585	0.784
length(item) = 11	0.587	0.783
length(item) = 12	0.594	0.784
length(item) = 13	0.594	0.783
length(item) = 14	0.596	0.784
length(item) = 15	0.599	0.784
length(item) = 16	0.602	0.787
length(item) = 17	0.602	0.787
length(item) = 18	0.604	0.786
length(item) = 19	0.603	0.786
SE(θ) < 0.3 or length(item) = 13	0.592	0.783
SE(θ) < 0.4 or length(item) = 13	0.584	0.782
SE(θ) < 0.5 or length(item) = 13	0.577	0.780
SE(θ) < 0.6 or length(item) = 13	0.553	0.767
SE(θ) < 0.7 or length(item) = 13	0.542	0.770
SE(θ) < 0.8 or length(item) = 13	0.524	0.758

*Note: None, no stopping rule (perform all items); sum score is the total raw score; $\hat{\theta }$ is estimated under the CAT-S; *r* is the Pearson correlation; *AUC* is the area under the ROC curve; High stress is based on the PSS sum scores.*

**FIGURE 2:**
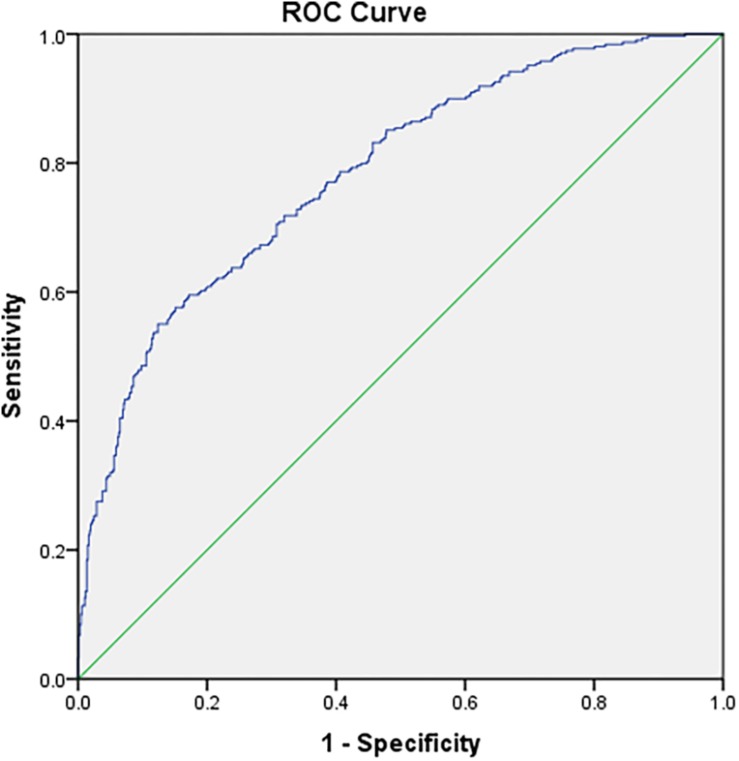
ROC curve for the stopping rule SE(θ) < 0.3 or length(item) = 13 in the stress CAT.

The third column of Table 4 shows the CAT’s diagnostic accuracy for the high stress classification in the AUC. The AUC values under all the rules were above the lower limit of moderate predictive utility (0.7). The diagnostic accuracy of the estimated θ based on the complete data was high and decreased as the level of measurement precision decreased. In addition, the ROC curve showed the relationship between sensitivity and specificity under the rule of “SE(θ) < 0.3 or length(item) = 13” in Figure 2.

## Conclusion and Discussion

As stress is one of the most prevalent phenomena in our daily lives, a simple and precise measurement of stress is necessary. However, many measures of stress have generally been based on CTT, and they were so time consuming that they increased the respondent burden. Therefore, efforts have been made to develop a short adaptive test (CAT-S) in this study.

In the construction of the item bank, items were removed by compromising on some statistical considerations, such as the assumption of unidimensionality, local independence, item discrimination, and DIF. The final item bank included 93 items that had good model fit, high discrimination, and no DIF and that met the assumption of unidimensionality.

After obtaining the final item bank, a simulated CAT program with different stopping rules was carried out to prove the efficiency of the CAT-S. The results demonstrated the following: (1) When the number of items was fixed, the average SE under each rule was above 0.322, and the reliability was higher than 0.890; also the correlation coefficient between CAT-S and the final stress item bank was greater than 0.937. (2) When considering both fixed length and termination accuracy, although the CAT-S administered only a small number of items, there was almost no difference between the amount of information provided by the CAT-S and the final stress item bank, and the stress levels estimated by the two tests were highly correlated. When the measurement accuracy was SE(θ) = 0.3, the test consisted of averagely 10.128 items, accounting for 10.89% of the final stress item bank. However, the correlation of estimated stress between the CAT and the total test was 0.937. Even if the measurement precision was reduced from 0.3 to 0.4, the participants were tested only on approximately 6.658 items, and the correlation coefficient was reduced only slightly, still as high as 0.915. (3) The level of reliability was high for the CAT-S: it was higher than 0.937 under the rules of fixed length, 0.895 under SE(θ) < 0.3, and even 0.849 when the measurement requirement was decreased to SE(θ) < 0.4, basing on a fixed length of 13. In addition, test and item reliability plots (see Figure 1) showed that the information from the CAT-S was almost 0.92 on the latent trait scale over −0.5, such that the CAT-S was a suitable measure for most participants. (4) The CAT-S has acceptable validity. There was a certain correlation between the CAT-S and PSS scales. When the measurement accuracy was set to 0.3 and 0.4 under the length of 13, the correlations between the stress estimated by the CAT-S and the total score of the PSS were 0.592 and 0.584, slightly less than the simultaneous validity (0.631) between the final stress item bank and the PSS scale. In addition, the ROC curve indicated that the diagnostic accuracy of the stress estimation was high. When the measurement precision was 0.3, the AUC (0.783) under the high-pressure classification was only slightly lower than the AUC of the final stress item bank (0.793). (5) When the fixed length was reduced, the measurement accuracy was also reduced. After the length was set to 13, the average number of administered items decreased as the required level of measurement precision decreased. Likewise, the relationship between the latent stress estimation using the full and adaptive assessment has almost become weaker, and the criterion validity of the latent stress estimation gradually decreased with the measurement error. (6) When the stopping rule was SE(θ) < 0.3 or length(item) = 13, the CAT-S had a better estimation for the college student population. Under this rule, most participants answered less items than the PSS, which ensured a high correlation with the stress estimation as measured by the final stress item bank, and the test had acceptable reliability and validity.

The main contributions of this study can be summarized as follows: (1) A CAT for measuring stress based on IRT was developed, and it met psychometric requirements such as unidimensionality, high discrimination, item fit, and no DIF. (2) The developed CAT-S was more efficient than the traditional P&P test under CTT. First, this adaptive approach can allow different people to take different tests, and the items can be chosen and administered differently. Second, the approach saves test time, which is equivalent to cost saving. (3) From the perspective of personality traits, considering that people with a strong sense of stress may not have the patience to complete many items (Furnham, 1997), the CAT-S can reduce this deficiency by answering fewer items. (4) Due to the function of the computer, the stress level of the participants can be continuously monitored, and the participants’ response time can be recorded easily through the theta estimates in CAT-S.

Some advantages of the CAT-S are as follows. First, different test score systems can be compared in the CAT-S. This approach allows the item difficulty to endorse and the participants’ stress level to be on the same scale. Second, the adaptive system adjusts for the item selection method accordingly, automatically selects the most appropriate next item for the participants, and finally estimates stress accurately. Third, the CAT-S has high efficiency and speed. Under the same conditions, only a small number of items were used to achieve high measurement precision, reliability, and validity, which greatly reduced the burden on the participants. Under the optimal termination rule [SE(θ) < 0.3 or length(item) = 13], 69.17% of the participants answered fewer items than the criterion scale (PSS) on the CAT-S, thus reflecting the superiority of the CAT. Finally, IRT accounts for parameter invariance, ensuring that the result will not be affected by other results regardless of whether the participant is in a representative sample. Therefore, the CAT-S can accurately, quickly, and efficiently assess the stress level of the participants.

However, certain limitations should be considered when drawing conclusions from this study. A CAT application on the Internet would make the procedure rather complicated, as the statistical algorithm would exchange information between computers and respondents. Extra items need to be included when measurement precision must be uniform over the latent trait scale. In addition, the CAT procedure was simulated in a statistical software environment by a computer using a special program. A real CAT should be performed to verify the reliability and validity in the near future. Furthermore, the test developers still have much to research because the CAT procedure must be carefully implemented and maintained.

In summary, this study outlined the steps of the development of the CAT-S and described its properties using real data. The CAT-S demonstrated the advantages stated and supported by theory in its early development stage. It results in an efficient and precise measurement of a highly relevant psychological construct. A large reduction in the number of items was obtained for the CAT-S; however, the reduction in items did not result in a relevant loss of test information. Notably, the CAT-S was found to have adequate reliability and validity. Moreover, the CAT-S was suitable for most participants on the latent trait scale over −0.5 for a large amount of information. There are fewer items with a lower location parameter, so participants with an estimated value below −0.5 may not be effectively measured. However, the value of this study is to better select high-stress people for intervention; for people with low stress, it is relatively unnecessary to pay too much attention to their low degree of stress, so the current results are acceptable. These results indicated that CATs are worth considering in order to improve psychometric assessment, but the evaluation of real CATs in practice should continue. We hope that these outcomes will be deemed useful and that CAT procedures can be used to measure latent variables such as stress in future studies.

## Data Availability Statement

The raw data supporting the conclusions of this article will be made available by the authors, without undue reservation, to any qualified researcher.

## Ethics Statement

The studies involving human participants were reviewed and approved by the Center of Mental Health Education and Research, School of Psychology, Jiangxi Normal University. The patients/participants provided their written informed consent to participate in this study.

## Author Contributions

XT designed the study and was responsible for the statistical analyses and the writing of the manuscript. BD provided ideas for programming, data analysis, and manuscript writing. All authors drafted the manuscript and approved it for publication.

## Conflict of Interest

The authors declare that the research was conducted in the absence of any commercial or financial relationships that could be construed as a potential conflict of interest.

## Footnotes

1 https://wenku.baidu.com/view/2e75e4074a7302768e993989.html

2 https://wenku.baidu.com/view/a12077980740be1e650e9ab9.html

3 https://wenku.baidu.com/view/2247919cda38376baf1fae72.html
